# Complete Revascularization of Reimplanted Talus After Isolated Total Talar Extrusion: A Case Report

**DOI:** 10.7759/cureus.7947

**Published:** 2020-05-03

**Authors:** Bandar N AlMaeen, Ibrahim S ElMaghrby, Mohammed K AlNour, Tareq A Alrefeidi, Saleh M Abu Adas

**Affiliations:** 1 Surgery/Orthopedic Surgery, College of Medicine, Jouf University, Al-Jouf, SAU; 2 Orthopedic Surgery, Prince Mutaib Bin Abdulaziz Hospital, Al-Jouf, SAU; 3 Miscellaneous, Jouf University, Sakaka, SAU; 4 Orthopedic Surgery, Armed Forces Hospital Southern Region, Khamis Mushyt, SAU; 5 Orthopedic Surgery, King Fahad General Hospital, Jeddah, SAU

**Keywords:** total talar extrusion, complete revascularization, early reimplantation

## Abstract

Total traumatic extrusion of the talus is a rare and disabling ankle injury. Treatment may include talar reimplantation or talar body removal, but an optimal treatment protocol has not yet been established. Several case reports showed that disruption of the vascular supply and contamination could lead to major complications, such as infection and avascular necrosis, with the high risk of these complications being associated with both the traumatic ankle injury itself and subsequent talar reimplantation. No report to date has described the revascularization of a completely extruded talus, as shown by serial MRI, a less invasive surgical strategy consisting of immediate reimplantation, early administration of antibiotics, and a short period of cast immobilization followed by early motion exercises. The present study describes complete revascularization and good clinical outcomes in a 30-year-old man who underwent talus reimplantation after isolated total talar extrusion.

## Introduction

Although rare, total traumatic extrusion of the talus is one of the most severe, disabling ankle injuries. Talus extrusion usually requires high-energy trauma and is often accompanied by severe soft-tissue damage, wound contamination, and fractures of the malleoli, talar body, and/or talar neck [1]. Total traumatic extrusion without a concomitant fracture is extremely rare, with rates estimated to be 0.06% of all dislocations and 2% of all talar injuries [2-3].

Treatment may consist of talar reimplantation or talar body removal, but the rarity of total talar extrusion has prevented the establishment of a treatment protocol [4-5]. According to several case reports, disruption of the vascular supply and contamination leads to major complications, such as infection and avascular necrosis. The high risk of these complications is associated both with the traumatic ankle injury itself and subsequent talar reimplantation [6-8]. Although early talectomy with tibiocalcaneal arthrodesis was once recommended as the initial procedure, reimplantation of a completely extruded talus through the wound has been recommended more recently, yielding satisfactory results [4-7].

We present a case of a patient who experienced open total traumatic extrusion of the talus and was successfully treated with immediate antibiotic administration, debridement, reimplantation of the talus through the open wound using intraoperative Schanz pins alone for manipulation and traction and cast immobilization with optimal clinical outcomes.

## Case presentation

**Figure 1:**
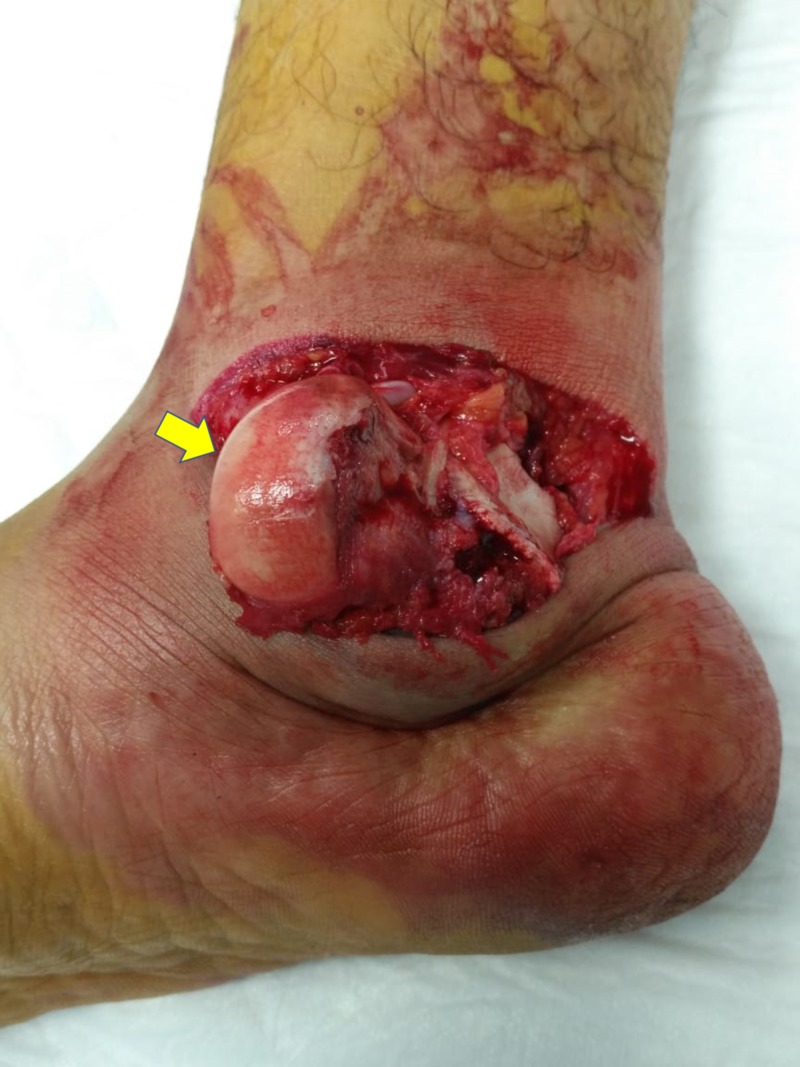
Clinical picture. Clinical presentation of the injured ankle with total talar extrusion.

A 30-year-old man presented to our ED by ambulance after being involved in a high-speed motor vehicle collision. On arrival, he was intubated and mechanically ventilated as he was unstable with a head injury. Initial physical examination revealed a visible deformity of the right lower extremity, with an approximately 10-cm laceration along the medial aspect of his ankle and a totally extruded and rotated talar body through the skin (Figure 1). The patient was diagnosed with a Type III open fracture and a right-sided total talar extrusion. The distal vascularity of his right foot was intact, and there were no associated fractures in both upper and lower limbs.

After closed reduction failed, the open fracture was initially irrigated with normal saline and splinted with sterile dressing. In addition, he was started on preventive antibiotics and given a tetanus toxoid booster. He was scheduled for open reduction and intraoperative irrigation as soon as possible.

His general condition did not allow initial radiographic and CT scanning of his extremities. Because the patient had a severe traumatic brain injury, with trauma brain CT showing an acute subdural and subarachnoid hemorrhage, he was taken immediately to the operating room to undergo an urgent decompressive craniotomy, and open fracture management and dislocation reduction. The patient was placed under general anesthesia and administered another dose of prophylactic antibiotics. After preparing and draping the patient's open wound, his extruded talus was cleaned and irrigated copiously with sterile normal saline. Devitalized tissues were debrided, with intraoperative radiological and clinical evaluation showing the talus was completely extruded from its articulation without any significant fracture. However, the inferior surface contained a small area of articular cartilage injury, loosely held by a few strands of the deltoid ligament, which were preserved. Excessive soft tissue stripping was avoided to prevent complete devascularization of the talus. Several trials were required to reimplant the extruded talus through the open wound. The first trial failed because the talus was blocked by stretching of the tibialis posterior and flexor hallucis longus tendons laterally over the neck of the talus. The tibialis posterior tendon was released to prevent reduction blocking, and a single Schanz pin was inserted from the medial to the lateral direction into the calcaneus for distraction, to open the ankle and subtalar joints and to maintain the gap of the joint surface (traction and counter traction technique). Another Schanz pin was inserted through the talar head to control the manipulation of the talus. Unfortunately, this Schanz pin became loose and reinserted in the talar body, which showed better manipulation of the talus. Reduction was achieved when the talar body was introduced initially, followed by the talar head by manipulation.

**Figure 2:**
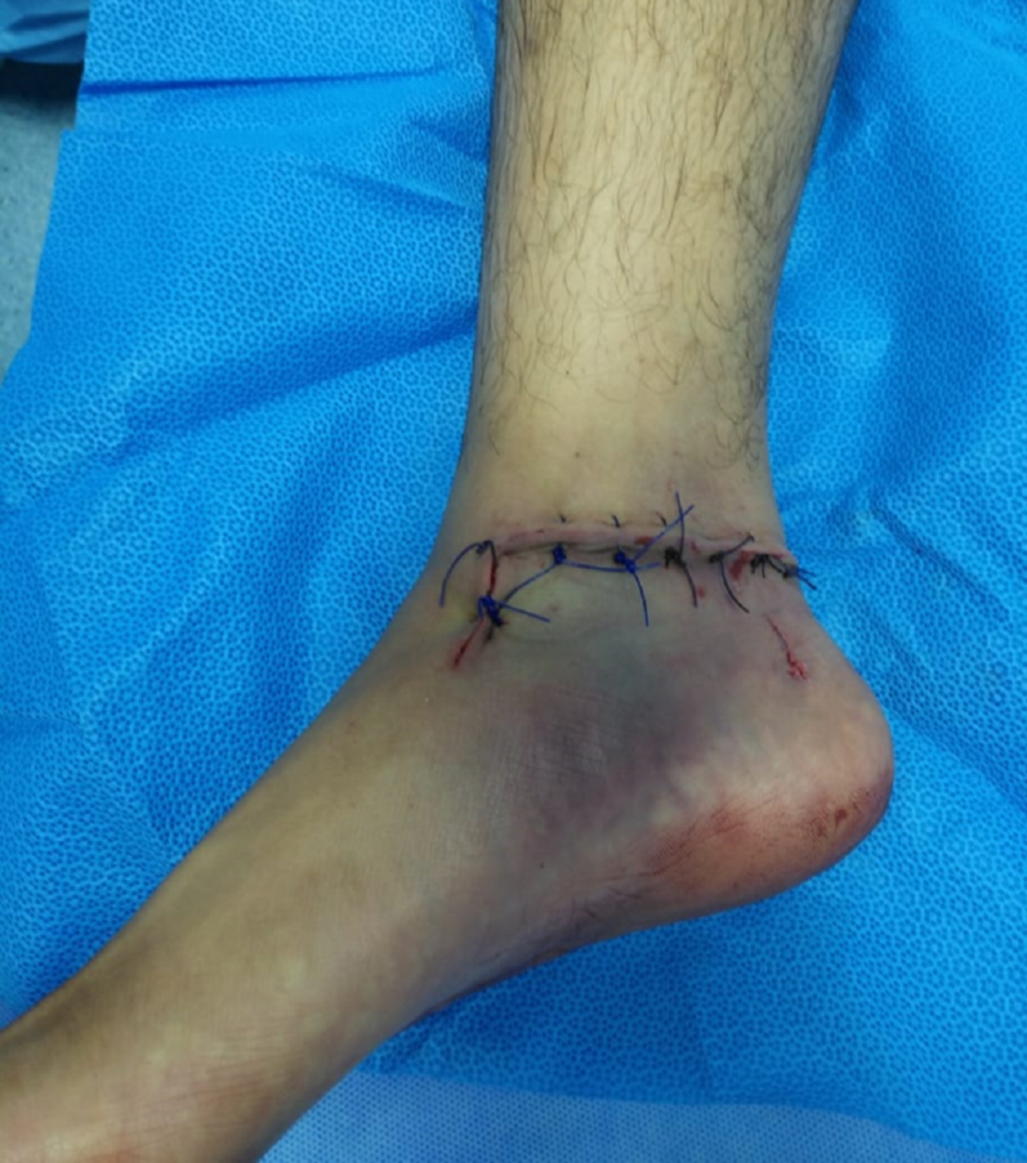
Photograph of the medial aspect of the right ankle after talar reduction, reimplantation, and wound closure.

In contrast, the reverse technique failed. The tibialis posterior tendon was repaired subsequently. Medial malleolus osteotomy was not needed and not performed after reduction. The ankle and subtalar joint examined were stable intraoperatively without inserting any Kirshner wires. After final irrigation with normal saline, the wound was closed, and sterile dressing applied (Figure 2). Repeat surgery was not required, as the wound remained clean and dry after dressings were changed in the ward.

**Figure 3:**
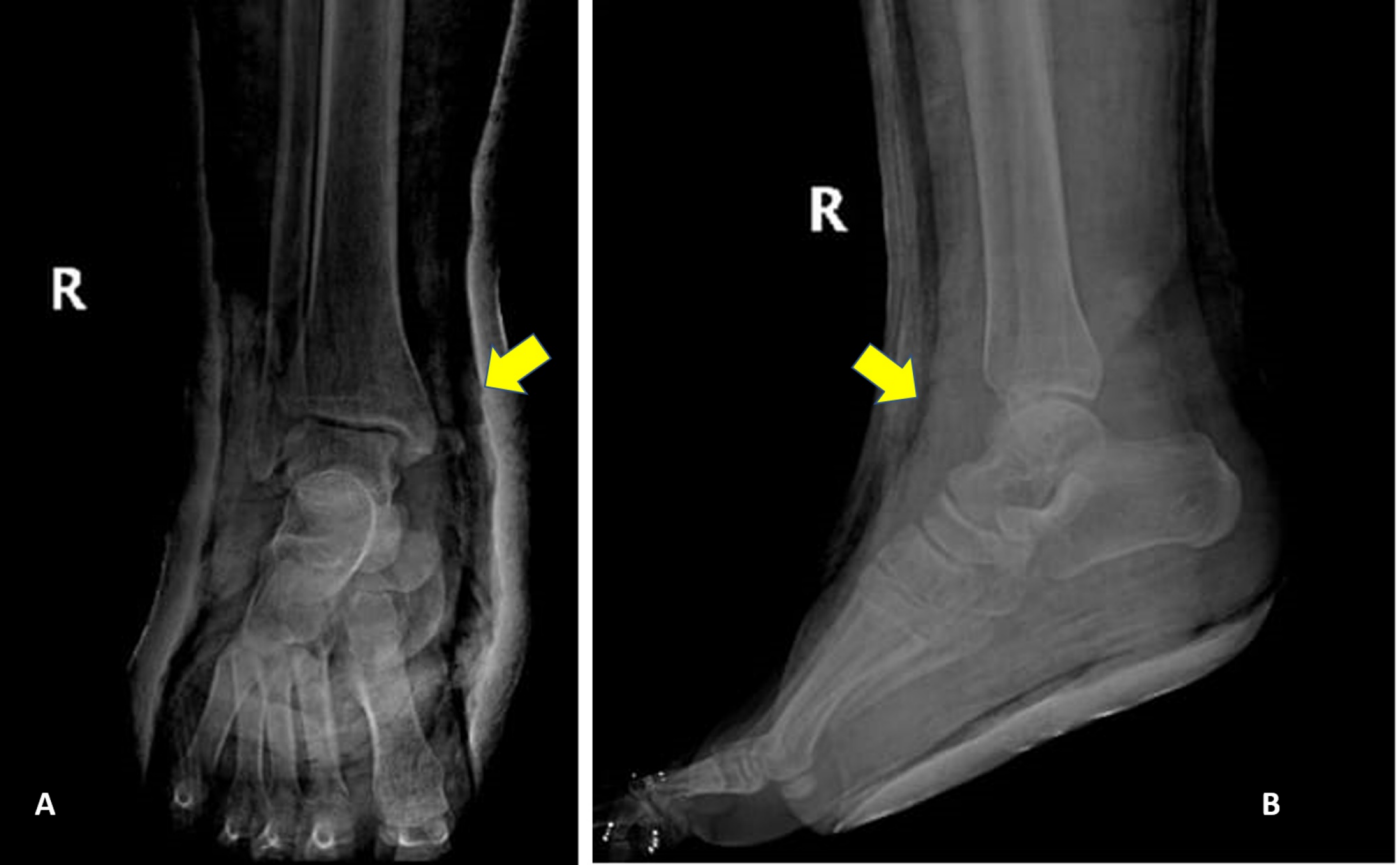
Postoperative (A) anteroposterior and (B) lateral radiographs showing the anatomical reduction of the ankle joint, including the subtalar and tibiotalar joints.

**Figure 4:**
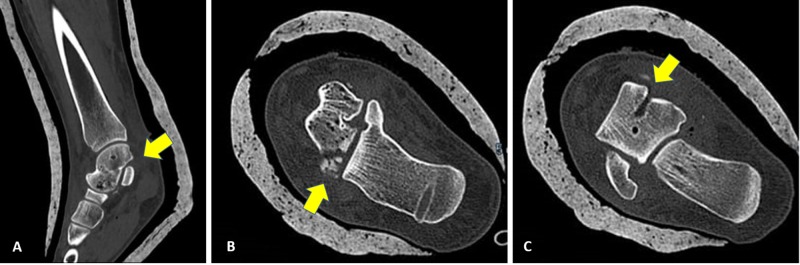
Postoperative (A, B) coronal and (C) sagittal computed tomography images showing the anatomical reduction of the ankle joint, including the subtalar and tibiotalar joints and small fragmentation involving the inferior surface of the talus. In addition, a small round defect was observed in the talar head and body, representing intraoperative sites of temporarily implanted Shanz pins.

Plain radiography (Figure 3A,B), and CT scans (Figure 4A-C) showed no evidence of talar neck or body fracture, with only a small area of fragmentation involving the inferior surface of the talus. Also, a small round defect appeared in the talar head and body, representing the sites of temporary intraoperative implantation of the Shanz pins.

Outpatient follow-up was routine; after six weeks, his cast was removed, and the wound healed without complication. The patient underwent a rehabilitative protocol to recover right ankle range of motion and muscle strength, with nonweight bearing strictly maintained. Two months after surgery, he was allowed protected weight-bearing using crutches; after three months, full weight-bearing was allowed. After four months, the patient was able to walk while bearing full weight without the use of an assistive device. No postoperative complications occurred.

**Figure 5:**
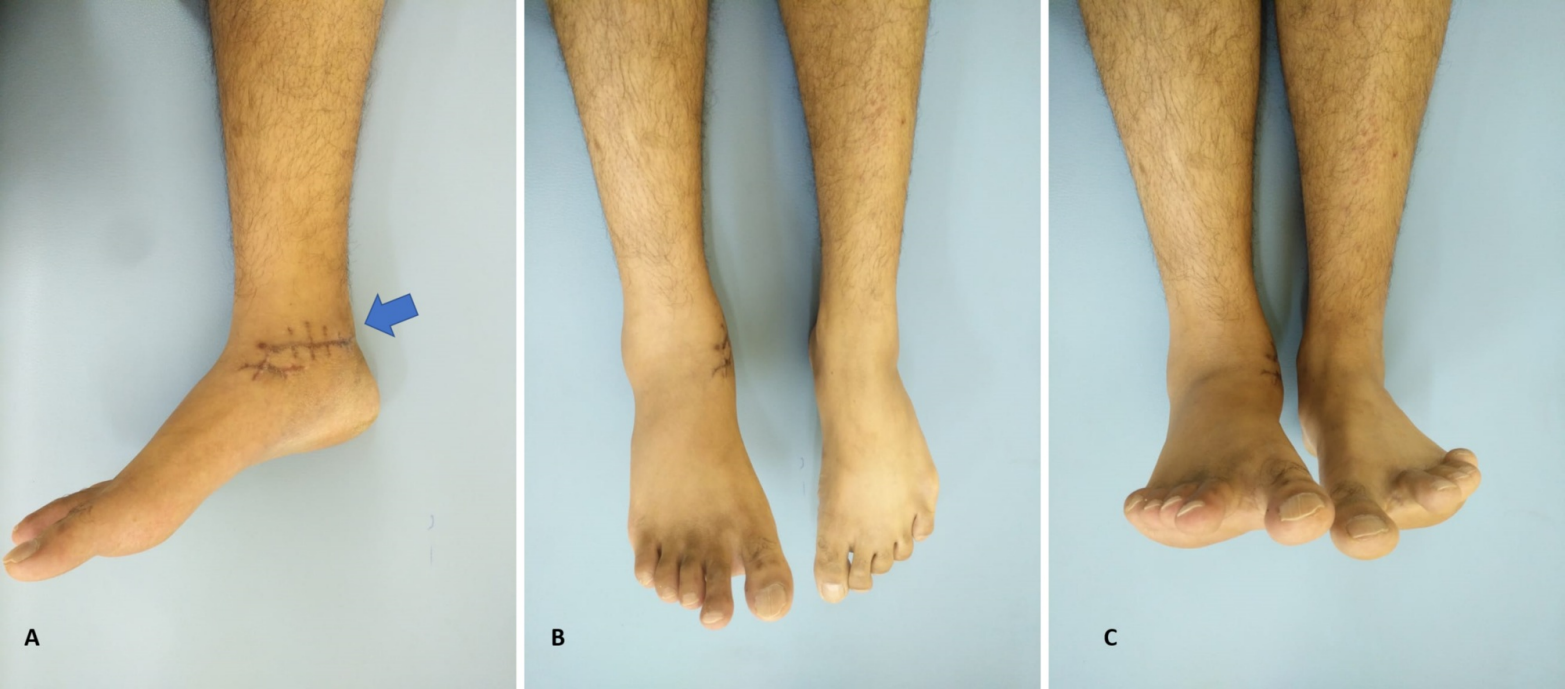
Clinical follow-up after 12 months showing the range of motion of the left ankle.

**Figure 6:**
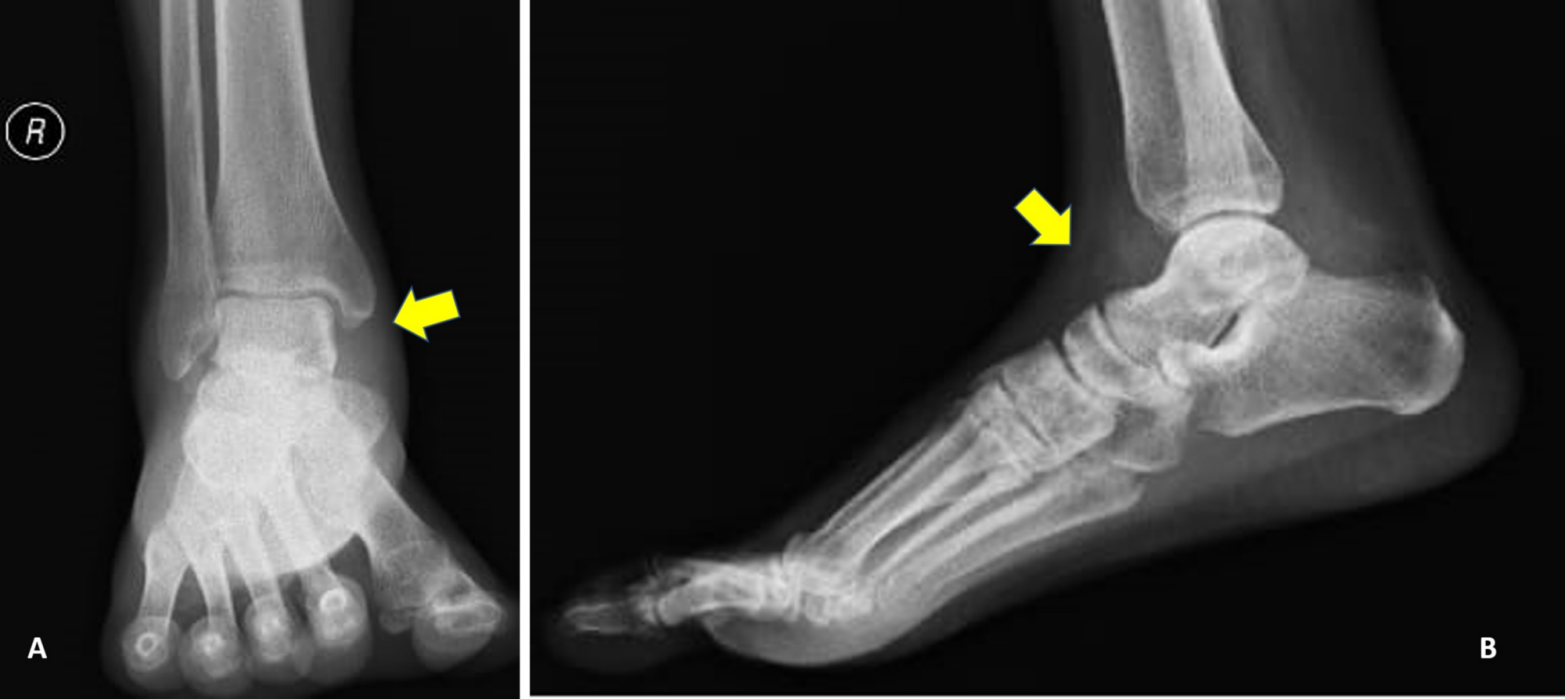
(A) Anteroposterior and (B) lateral radiographs of the ankle at 12 months.

**Figure 7:**
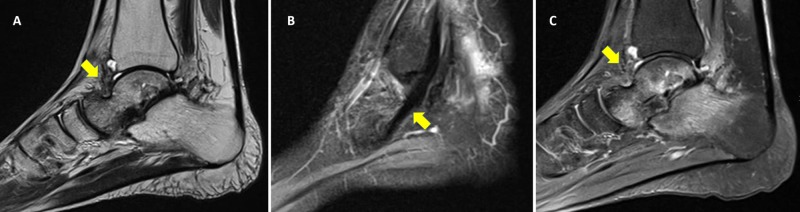
Non-contrast magnetic resonance images after one year. (A) T1-weighted image and (B) T2-weighted images, showing no signs of avascular necrosis or tibiotalar arthritis. (C) Sagittal T2-weighted image showing a healed intact tibialis posterior tendon.

Approximately one year after surgery, the patient reported no pain with weight-bearing and range of motion of the right ankle. A physical examination showed 30° plantar flexion and >15° dorsiflexion of the right ankle, markedly greater than the dorsiflexion observed in the contralateral extremity. He also displayed unrestricted subtalar joint motion (Figure 5A,C). Plain radiographs showed no evidence of increased radiodensity of the talus, with a viable talus reduced in the ankle mortise. Some mild posttraumatic changes were noted more prominently in the subtalar joint (Figure 6A,B). Noncontrast sagittal MRI showed normal T1 marrow signal involving the talar body, neck, and proximal head when compared with the marrow signals of adjacent calcaneus and navicular, results consistent with talar revascularization (Figure 7A). Sagittal T2-weighted imaging showed a healed intact tibialis posterior tendon with reactive edema in the subtalar portion of the calcaneus and tibial plafond. Again marrow signals in the talar body and neck were normal (Figure 7B,C). The patient experienced no complications during his one-year follow-up, with no evidence of increased sclerosis of the talar body, no narrowing of tibiotalar joint spaces, and no signs of avascular necrosis. The patient could walk without aids, pain-free.

## Discussion

Open total talus extrusion without associated fractures is a rare injury. To our knowledge, the largest published series to date include 18 and 27 ankles [7, 9]. These injuries are usually caused by high-energy trauma, such as a fall from a height or a motor vehicle injury. Tibiotalar or subtalar dislocations may be associated with malleolar fractures until the talus is completely extruded laterally [6, 10].

Talectomy with immediate tibiocalcaneal arthrodesis was usually favored over reimplantation arthrodesis for an open total talar dislocation [11-12]. Recently, however, reports of favorable outcomes with reimplantation suggest this method as a first-line treatment [13]. Prompt reduction and salvage of the extruded talus is considered a relatively safe and effective procedure [10, 14-15].

Infection and avascular necrosis (AVN) are the most important complications associated with open talar extrusion. Anatomically, 60% of the talus is covered with articular cartilage, with no muscular attachments, making the talus vulnerable to dislocation. Extreme supination and plantarflexion forces cause dislocation of the talus from the ankle mortise, disrupting the strong ligamentous attachments causing open injury [16].

Blood is supplied to the talus by an intricate arrangement of blood vessels that are highly vulnerable to injury [17]. The vascular supply to the talus consists of the anterior tibial, posterior tibial, and perforating peroneal arteries. The artery of the tarsal canal, a branch of the posterior tibial artery, supplies most of the talar body, the medial talar wall, and the undersurface of the talar neck. The artery of the tarsal canal anastomoses with the artery of the sinus tarsi, which is a branch of the perforating peroneal artery, and these vessels supply the inferior aspect of the talar body and neck [18]. Dislocation of the talus from the ankle mortise results in sequential failure of talar blood supply. Moreover, total talar dislocation and extrusion results in the total disruption of the talar blood supply and a high risk of vascular crisis. The risk of AVN was highest when no soft tissues remained attached to the talus [16]. In the early postoperative phase, the development of AVN is very difficult to predict. AVN is usually observed from six months to two years after the injury. Hawkins' sign (consisting of subchondral radiolucency in the talar dome six to eight weeks after the injury-indicative of early subchondral atrophy) is the only early predictor of revascularization that can be seen on conventional radiographs [12]. MRI remains the most specific and sensitive method of detecting early development of AVN [10].

Studies have reported that times between talar exposure and reimplantation have ranged from 30 min to eight days and that increased risks of osteomyelitis and AVN are associated with increased time to reimplantation [4, 6, 8, 10, 16]. Generally, prognosis is favorable if reimplantation is performed within three hours, but is poor and associated with secondary tibiocalcaneal arthrodesis when patients are treated >24 hours after injury. During the 12-month follow-up period, our patient experienced none of the major complications associated with total talar dislocation.

The incidence of infection can be decreased with the use of a proper open fracture protocol and careful soft tissue handling [9]. If the wound is severely contaminated or the time to reimplantation delayed, talectomy and tibiocalcaneal arthrodesis must be considered [16]. Only one of 27 open talar extrusions treated over nine years became infected during the initial period of hospitalization [9]. The low infection rate was ascribed to staged procedures, multiple debridements, early soft tissue closure, and rigid fixation. Recent recommendations include preservation of the talus except in the case of gross contamination [16]. A report on 18 open injuries of the talus, 12 of which were partial or total talar extrusions, found that the overall infection rate was 38%, with greater soft tissue injury associated with an increased prevalence of infection [7].

Fixation constructs for a reimplanted talus have included external fixation without talar fixation [4, 10], and insertion of two Steinmann pins from the inferior aspect of the calcaneus through the talus into the distal tibia with a circular external fixator [2]. Another fixation construct was made of two retrograde Kirschner wires [1]. Their final outcomes were not clear as the postoperative radiographs were not included and no strategy for the use of fixation constructs has been established.

To our knowledge, no report to date has described the revascularization of a completely extruded talus, as shown by serial MRI, without any method of fixation. Our findings revealed the possibility of revascularization with less invasive surgical strategy consisted of immediate reimplantation, early administration of antibiotics, and a short period of cast immobilization with early motion exercises. This protocol may prevent additional damage to soft tissue around the ankle joint and limb disability due to prolonged immobilization. In our patient, the total talar extrusion was managed by early reimplantation and the minimization of soft tissue damage. This approach yielded an excellent outcome and complete revascularization after one year.

## Conclusions

Open total talar extrusion is a very rare, challenging injury, with few reported cases in the literature. Primary talus reimplantation is the treatment of choice, preserving good ankle function, normal joint anatomy, and avoiding further reconstructive procedures. Early reimplantation using proper reduction techniques, administration of antibiotics, preservation of remaining viable soft tissue, early soft tissue closure, and early postoperative rehabilitation can enhance patient prognosis.
